# Acute Pyelitis in Infancy

**Published:** 1912-07

**Authors:** Edith R. Hatch

**Affiliations:** 2565 Main Street, Buffalo; 2620 Main Street


### BUFFALO MEDICAL JOURNAL

Vol. Lxvii. JULY, 1912. No. 12

### Acute Pyelitis in Infancy

By DR. EDITH R. HATCH,
2565 Main Street, Buffalo

THE present day literature abounds with contributions on diagnoses or urinary disorders with cytoscopy and ureteral catheterization. The majority of general practitioners must needs barely glance at the titles of these lengthy articles as time forebears of their reading. Also, one feels that much therein stated is possible only for the specialist to carry out.

It is not with the idea of bringing forth anything new on this subject that this paper is presented but rather to emphasize and urge better recognition of *one* urinary disturbance which comes to the general practitioner, and the diagnosis of which necessitates of no new or special mechanism. In selecting the subject Acute Pyelitis of Infancy the writer purposely avoided substitution of the word childhood for infancy, having in mind the babe under two years of age—the age when, unless the diagnosis be that of marasmus or intestinal disturbance, the physician often mentally agrees with the worried mother that "it is so hard to tell what is the trouble with a baby for he can't talk."

Just as the general practitioner cannot carry out all the new mechanical devices and intricate laboratory tests neither can he read all the journals—he must principally rely upon text books of general diagnosis with a few selected ones on special subjects. Therefore, in the average library there will probably be found very little space or accurate information upon this subject.

In the 1896 edition of Ashley and Wright's Diseases of Children there is this paragraph: "Acute Pyelitis is certainly not a common disease in infants or children. We have however several cases of acute illness in infants or young children accompanied by a high temperature of an intermittent type and after the attack lasted several days it has been noted that the urine contained pus—the nurse having called attention to the fact that there was something unusual in the way the urine stained the diapers. Dr. S. J. Gee has recorded a similar case in an infant of 9 months. Dr. Emmett Holt records three such cases in infants of 8, 9 and 14 months."

Dr. Holt's 1897 edition takes up at some length the definition, etiology, lesions, symptoms, diagnosis, prognosis and treatment of pyelities, considering it as a secondary affection but mentioning these same three cases in which it was apparently a primary affection. Jacobi, Rotch, Still, Morse and Koplic at this same period and for some time later refer to it as a very rare malady. In fact the first case reported by Escherich was not until 1894,

Holt no longer counts the number of cases seen, and in fact all the writers at the present time are united in the opinion that acute pyelitis, or inflamation of the mucous membrane lining the pelvis of the kidney, is *not* a rare disease. This concensus of opinion among pediatrists is quite in contrast to the diversion of opinions still waging concerning other problems as for instance infant feeding.

Let us first consider the clinical picture of this complaint from an actual typical case. D. B., girl, 8 months old, previously well, bottle fed with modified cow's milk. Mother thought child unduly hot and herself took the temperature at 5 P. M. and it was 105 by rectum. When seen a few hours later this was confirmed. Of course the symptoms are objective in patients of this age. There was no history of vomiting but food had not been taken as well as usual; no diarrhoea; no suppression of the urine. Physical examination of the chest and abdomen showed nothing abnormal—no distension of abdomen or tenderness over kidneys or bladder. There was no redness of the tympanic membrane or tenderness over mastoid region. The mother had already given calomel which was followed with milk of magnesia. The child was quickly gotten into a profuse perspiration and temperature fell to 101 before midnight. The next morning it was still 101 but reached 103 in the afternoon. This irregularity ranged from 100 to 105.4 for eight days, the child looking decidedly ill, pale and frequently refusing food. At no time was there vomiting, painful or frequent urination, but for the first few days the urine was thought to be somewhat scanty. While the usual suspicion would be of an intestinal derangement at no time would an examination of the stools have confirmed it. A sample of urine was collected the first night and found to be cloudy, acid reaction, specific gravity 1010, slight trace of albumin, pus cells in quantity, no casts or red blood cells. Diagnosis acute pyelitis and treatment as hereinafter mentioned. The temperature fell to normal in 8 days. Iron was given for the subsequent anemia and there has been no recurrence in the last year and a half.

We may ask what in the foregoing may be considered significant of pyelitis—nothing, excepting to be suspicous. In any obscure irregular temperature *always* examine the urine and ears.

If diagnosis still remains obscure of course the blood should be examined for typhoid and malaria. However, the *urinary* findings *are* most significant—acid reaction, low specific gravity, none of slight trace of albumin, microscopically many pus cells, singly and in clumps, the amount of the albumin present being only in proportion to the pus, epithelial cells and occasionally a red blood cell. The pus will vary in amount which is the reason for examining more than one sample if the first ones be clear.

While chills are so pronounced in older people they are rarely seen in infants or at least much less severe.

Failure in diagnosis is often probably due to the idea that it is so difficult to collect urine from an infant. While in the boy it is comparatively easy with a large test tube or wide mouth bottle held with adhesive straps it seems much more difficult with the girl. There are several special devices for the girl, but after they are all tried this same wide mouth bottle will be found as satisfactory as any, and with a little extra watchfulness an uncontaminated sample can be promptly obtained. About the neck of the bottle buttonhole a piece of adhesive having a slit in the posterior part—one tail for each buttock. This can be fitted on closely and held in place by the diaper. Supporting the buttocks on a pillow also aids. To wait for the chance of obtaining sample by passing in a vessel generally means delay of several hours or utter failure unless the child has already been trained for its use. If the family is absolutely unable to manage this a catheterized specimen should be obtained, which is especially easy in girls.

Happily these babes are already on fairly good treatment in spite of no diagnosis or an incorrect one as they are milk fed and probably given extra water because of the fever. Also, they seldom prove fatal. They either recover spontaneously or become chronic. The treatment consists of milk diet with plenty of water and alkalies. 15 to 25 grains of potassium citrate or ammonium acetate should be given in 24 hours to a year old babe. While this is generally sufficient urotropin apparently hastens recovery. If the case does not respond an autogenous vaccine should be used before it becomes chronic.

Eighty per cent or more of the cases occur in girl babies. Because of this and because the colon bacillus is usually found as the germ it appears likely that it is an ascending infection. Mothers and nurses should be instructed to cleanse the napkin parts with cleans pieces of cotton, never wiping *forward* over the perineum. As the virulence of the colon bacillus is markedly increased in diarrhoea and other intestinal diseases special precaution should then be exercised as it is sometimes secondary to diarrhoea.

The majority of the books state that the colon bacillus does not produce leucocytosis but the white count in these cases frequently runs as high as 15-18,000.

It is interesting to note that these cases are not associated with vulvo-vaginitis and that the mucous membrane of the urinary tract of an infant seems to resist the gonococcus better than the colon.

So unusual is it to find primary pyelitis in males that the following case is cited. E. A., boy, colored, 9 months, breast fed, obscure irregular temperature for a week. Urine was slightly cloudy, acid, no albumin, no pus but even a catherized sample swarmed with bacteria. The urine was frequently examined expecting pus might appear. The ears were never inflammed. Because the patient was a boy it was thought *probable* that it was *not* pyelitis but the urine was cultured, and in the meantime the blood was often examined. The white count ran about 7,000, no malarial organisms, and typhoid was negative until the tenth day when there was agglutination and the disease ran a typical course, impressing upon one that obscure, high and irregular temperature with bacilluria is not sufficient to establish a diagnosis of acute primary pyelitis—that the urinary findings *must* include pus cells, and that infants are liable to infections such as typhoid, although rare.

2620 Main Street.

